# MicroRNAs overexpressed in Crohn’s disease and their interactions with mechanisms of epigenetic regulation explain novel aspects of Crohn’s disease pathogenesis

**DOI:** 10.1186/s13148-021-01022-8

**Published:** 2021-02-18

**Authors:** Cecilia Fernández-Ponce, Roberto Navarro Quiroz, Anderson Díaz Perez, Gustavo Aroca Martinez, Andrés Cadena Bonfanti, Antonio Acosta Hoyos, Lorena Gómez Escorcia, Sandra Hernández Agudelo, Christian Orozco Sánchez, José Villarreal Camacho, Linda Atencio Ibarra, Jose Consuegra Machado, Alberto Espinoza Garavito, Francisco García-Cózar, Elkin Navarro Quiroz

**Affiliations:** 1 Department of Biomedicine, Biotechnology and Public Health, University of Cadiz, Cadiz, Spain; 2 CMCC-Centro de Matemática, Computação E Cognição, Laboratório do Biología Computacional e Bioinformática–LBCB, Universidade Federal Do ABC, Sao Paulo, 01023 Brazil; 3 Facultad de Ciencias Básicas y Biomédicas, Universidad Simon Bolivar, 080001 Barranquilla, Colombia; 4 Universidad Rafael Nuñez, 130001 Cartagena, Colombia; 5 Department of Nephrology, Clinica de La Costa, 080001 Barranquilla, Colombia; 6 School of Medicine, Universidad Libre, 080001 Barranquilla, Colombia; 7 School of Medicine, Universidad Simon Bolivar, 080001 Barranquilla, Colombia; 8 Centro de Investigación E Innovación en Biomoléculas, C4U S.A.S, 080001 Barranquilla, Colombia

**Keywords:** MicroRNA, Crohn’s disease, Epigenetic mechanisms, Post-translational modifications, T cells

## Abstract

**Background:**

In this review, we were interested to identify the wide universe of enzymes associated with epigenetic modifications, whose gene expression is regulated by miRNAs with a high relative abundance in Crohn's disease (CD) affected tissues, with the aim to determine their impact in the pathogenesis and evolution of the disease.

**Methods:**

We used HMDD and Bibliometrix R-package in order to identify the miRNAs overexpressed in CD. The identified enzymes associated with epigenetic mechanisms and post-translational modifications, regulated by miRNAs upregulated in CD, were analyzed using String v11 database.

**Results:**

We found 190 miRNAs with great abundance in patients with CD, of which 26 miRNAs regulate the gene expression of enzymes known to catalyze epigenetic modifications involved in essentials pathophysiological processes, such as chromatin architecture reorganization, immune response regulation including CD4+ T cells polarization, integrity of gut mucosa, gut microbiota composition and tumorigenesis.

**Conclusion:**

The integrated analysis of miRNAs with a high relative abundance in patients with CD showed a combined and superimposed gene expression regulation of enzymes associated with relevant epigenetic mechanisms and that could explain, in part, the pathogenesis of CD.

**Supplementary Information:**

The online version contains supplementary material available at 10.1186/s13148-021-01022-8.

## Background

Crohn’s disease (CD) is a recurrent inflammatory bowel disease (IBD) that is increasing in prevalence worldwide. CD is thought to result from a combination of genetic, immune and environmental factors. In recent years, the intestinal microbiota has also been shown to play a role in the development of the pathology [1–3]. These factors can lead to inappropriate chronic activation of the mucosal immune response, often resulting in strictures or fistulas [4].

To obtain a better understanding of the underlying mechanisms of CD, it is important to investigate the molecular and genetic changes involved in the onset and chronification of the disease. Studies of gene expression have been carried out jointly in patients with CD to identify gene expression profiles associated with adaptive immune response, cell adhesion, barrier integrity and extracellular matrix remodeling [3, 5–7]. Gene expression is partly regulated by microRNAs (miRNAs), small non-coding RNAs that bind to the 3′ untranslated regions of target mRNAs and negatively regulate their stability or translation [8]. miRNAs have been found to be involved in several pathologies, such as cancer, autoimmune disease, inflammatory disease including IBD, among others [9, 10]. Some studies focused on understanding the role of miRNAs in IBD, have described different miRNA expression profiles in patients with active or inactive disease, comparing with normal controls [11]. In CD, it has been demonstrated that single-nucleotide polymorphisms (SNPs) in miRNA binding sites may affect the expression of target mRNAs that are involved in the pathogenesis of the disease. In this regard, Brest et al. have shown that a synonymous variant in IRGM gene affects a binding site of miR-196, resulting in deregulation of IRGM-dependent xenophagy in CD [12, 13]. Other researches have evidenced the association of specific IL-23R gene variant with IBD susceptibility, which could be explained by the IL-23R gene variant inability to bind to miRNAs Let-7e and Let-7f. The loss of miRNA regulation induces high levels of both IL23R mRNA and protein production, and it could contribute to disease chronification, through sustained IL-23R signaling [12–14]. Thus, genetic research in IBD has made possible to identify several genetic factors; however, these factors only explain a small proportion of IBD complexity and variants. Epigenetic modifications of the genomic landscape studies and their association with identified genetic factors, open an interesting scenario that could explain the interactions between genes and environment, displaying new knowledge about IBD pathogenesis [15].

Epigenetic modifications are defined as heritable changes in gene expression that are mitotically and/or meiotically heritable and that do not involve a change in DNA sequence [16]. Thus, epigenetics has a great influence in gene expression and function generating varieties in cell differentiation, morphogenesis and adaptability of an organism. These findings suggest that epigenetic mechanisms could connect genotype and environmental factors and thusly lead to phenotypical changes [17]. Epigenetic modifications, such as DNA methylations and histone modifications, have been evidenced to be implicated in carcinogenesis, psychiatric diseases, neurodegenerative disorders, autoimmune and chronic inflammatory diseases [10, 15–21]. Regarding this matter, several studies have demonstrated the association of IBD susceptibility and pathogenesis with alterations in the methylation status of IBD-associated genes. DNA methylation generates changes in gene expression levels involved in immunological response regulation pathway, intestinal epithelial barrier integrity, stricture formation, among others, implicated in IBD pathogenesis [15, 22]. According to these findings, it is interesting to mention that in patients affected by ulcerative colitis (UC), an increased DNA methylation of Runt-related transcription factor 3 (RUNX3), Protease activated receptor 2 (PAR2) and E-cadherin genes have been found [23–25]. Additionally, colonic inflammation in IBD patients has shown association with colonic mucosal microbiota composition and DNA methylation patterns [26].

Histone methylation and acetylation have been less extensively studied in IBD. In ileal epithelial cells of newly diagnosed pediatric CD patients, it has been described an histone methylation pattern characterized by increased histone H3-lysine 4 trimethylation (H3K4me3) affecting genes involved in cytokine functions and epithelial immune response and decreased H3K4me3 shown by genes associated with metabolism [20].

Regarding histone acetylation, in several human cancers, including colorectal carcinomas, histone deacetylases (HDACs) expression levels have been found increased [27–30]. Interestingly, in experimental colitis, HDAC inhibitors reduce colitis severity, suppress the secretion of pro-inflammatory cytokines, expand the number of Foxp3+ Regulatory T cells (Tregs) and enhance their suppressive function [31–33]. These findings demonstrate that alterations in histone acetyl-transferases (HATs) and HDAC expression are not only involved in neoplastic development, but also in inflammatory phenomenon. On this basis, it has been shown that the short chain fatty acid (SCFA) butyrate, an HDAC inhibitor produced by colonic commensal bacteria fermentation of dietary fibers, induces in naïve CD4 + T cells, an increasing of histone H3 acetylation in the promotor and conserved non-coding sequence regions of Forkhead box P3 (Foxp3) locus, promoting their differentiation to Tregs [34, 35]. Additionally, propionate, another SCFA generated from gut bacterial fermentation, induces in colonic Tregs, an increased regulatory phenotype characterized by elevated Foxp3 expression and high secretion of the anti-inflammatory cytokine IL-10. Propionate improves the immunosuppressive functions of colonic Tregs by decreasing HDAC6 and HDAC9 levels, thereby enhancing histone acetylation, in a G-protein-coupled receptor-mediated process [36]. Furthermore, in dendritic cells (DCs), the HDAC inhibitory activity of butyrate, downregulates Relb, transcription factor required for the activation and function of DCs, and induce a DCs phenotype that promotes the expression of Foxp3 in CD4+ T cells and therefore Treg cell differentiation [34]. Thus, these findings show that the SCFAs, metabolites generated in high amounts by the intestinal microbiota, are capable of regulating the gut immune balance between pro- and anti-inflammatory processes via epigenetic mechanisms. In this regard, several studies show the relevance of the diet high in fiber and low in saturated fats to maintain the balance of the intestinal microbiota [37].

In this regard, analysis of gastrointestinal mucosal-associated microbioma from IBD patients samples has shown a remarkable dysbiosis characterized by a decrease in butyrate-producing bacteria species, such as *Roseburia intestinalis, Faecalibacterium prausnitzii, Eubacterium rectale* and *Butyricicoccus pullicaecorum;* and an increase in species such as *Escherichia coli, Fusobacterium nucleatum, Haemophilus parainfluenzae*, among others involved in inflammatory and tumorigenesis processes [38, 39]. Likewise, the SCFAs concentration, in fecal samples of IBD patients, is also diminished, mainly the butyrate and acetate levels, in patients with active UC [40–42]. The etiology of dysbiosis in IBD patients is not clearly understood, but it is known that the IBD patients show an immunological response against the intestinal microbiota that induce a loss of the intestinal barrier integrity and the consequently microorganism infiltration. These events contribute to the dysbiosis developing, the inflammatory response exacerbation and the injury of the gastrointestinal mucosa [43]. Thus, dysbiosis in the gut commensal microbiota composition, via epigenetic regulation mechanisms, has a role not only in IBD pathogenesis, but also as a risk factor for the development and progression of the disease.

Interestingly, epigenetic mechanisms have been evidenced to regulate gene expression including miRNAs expression, and likewise, miRNAs has shown to affect epigenetic mechanisms, by targeting genes encoding enzymes, such as DNA methyltransferases (DNMTs), HDACs and Histone methyltransferases (HMT). Thus, a feedback loop regulates miRNAs and epigenetic mechanisms interactions [16, 44–46]. On this subject, some authors have found that several miRNAs, associated with physiological and pathological conditions, can control gene expression establishing, promoting and regulating epigenetic mechanisms. Regarding physiological processes, miR29 and miR140 have shown a role in embryogenesis mediated by epigenetic modifications. Tuddenham et al. have described the HDAC4 as a target of miR-140, a miRNA expressed in cartilage tissues during the mouse embryos development [47]. Otherwise, miR-29b has been implicated in DNA methylation processes through the regulation of Dnmt3a/3b and Ten-Eleven Translocation methylcytosine dioxygenases Tet1/2/3 gene expression, during porcine embryogenesis [48]. Furthermore, the downregulation of DNMT1 by miR-377 overexpression has been associated with senescence and with alterations in the promoter methylation level of various genes as the Tumor Suppressor Gene p53 [49]. In tumorigenesis, low level of miRNAs as miR-137, whose target gene is the histone demethylase KDM5B, is associated with the increased expression of KDM5B and with cell proliferation [50]. In the same way, the Histone-Lysine N-Methyltransferase EZH2 is a target of miR-101. In Bladder carcinoma cells, miR-101 is found in reduced levels correlating with high expression of EZH2 and phenotypically, with tumor growth [51]. Additionally, in tumor-associated endothelial cells, VEGF is involved in mir-101 downregulation and consequently, the EZH2 expression increases promoting angiogenesis [52]. Likewise, in colorectal cancer (CRC), including IBD‑associated CRC, the enzymes DNMT3A and DNMT1 evidenced as targets of mir-143 and miR-342, respectively, have shown increased expression levels, correlating with low levels of miR-143 and miR-342 and thereby with tumor growth and cell proliferation [53, 54]. Thus, the role of the regulation of DNA methylation and histone acetylation by miRNAs as miR-185, miR-221, miR-152, miR-200a, among others, has been related to alterations in gene expression of several genes associated with tumorigenesis processes [55–58]. Regarding infection diseases, it is interesting to mention that the Hepatitis B virus X protein induces low expression levels of miR-101 and thereby alterations in DNA methylation due to the upregulation of its target gene DNMT3A. These findings are related to carcinogenesis and suggest the role of viral proteins in oncogenesis via alterations of miRNAs expression associated with epigenetic mechanisms [59]. Related to chronic inflammatory conditions and autoimmune disease, miR-34a has a role in atheroma plaque formation, through the regulation of HDAC1 in Hyperhomocysteinemia condition [60]. In Systemic Lupus Erythematosus (SLE), the association of miRNAs dysregulation with T cells phenotype alterations has been explained by variations in the expression of their target genes involved in epigenetic mechanisms, such as DNMTs and EZH2 [10, 21, 61].

Therefore, characterization of the roles of epigenetics and miRNAs in CD, and identification of their interactions, could provide useful knowledge which will offer a realistic and practical platform in the generation of predictive diagnostic tests, therapies and monitoring. The aims of the present study are to identify in silico the relationship among miRNAs and genes encoding enzymes associated with epigenetic and post-translational modifications in CD and to determine their impact in the development of the disease and the susceptibility to complications.

## Methods

### Identification of miRNAs found overexpressed in CD patients’ tissues

miRNAs associated with CD were identified using HMDD databases and original papers from Web of Science and Pubmed. The search terms used were “Crohn’s disease” and “microRNAs.” The searching was performed with the help of the R Bibliometrix package.

### Selection of miRNAs found overexpressed in tissue samples from CD patients that regulate enzymes associated with epigenetic and post-translational modifications

In order to identify miRNAs overexpressed in CD-affected tissues and that regulate the expression of enzymes associated with epigenetic and post-translational modifications, 110 papers that reported the upregulation of 190 miRNAs in CD patients were cross-indexed with 1,544 miRNAs that interact with mRNAs for enzymes related to epigenetic and post-translational modifications. To identify miRNAs that regulate epigenetic and post-translational modifiers was used mirPath v.3 (acetylation, bromination, formylation, gamma-carboxyglutamic acid, hydroxylation, lipoylation, LTQ, methylation, N-linked glycosylation, N6-carboxylysine, nitration, O-linked glycosylation, oxidation, phosphorylation, pyridoxal phosphate, pyrrolidone, carboxylic acid, pyruvate, retinal protein, S-nitrosylation and sulfation). The identified miRNAs-target mRNA interactions are validated from specific, as well as high-throughput experiments. The results obtained from both databases were compared to obtain the miRNAs dataset associated with epigenetic and post-translational modifications.

### Construction of functional interaction network of enzymes associated with epigenetic and post-translational modifications, whose expression is regulated by miRNAs overexpressed in CD

Protein–protein biological interaction of identified enzymes associated with epigenetic and post-translational modifications and regulated by miRNAs overexpressed in CD, were analyzed using String v11 database. Physical as well as functional interactions with medium confidence of 0.4 were analyzed in order to perform the interactive network. In addition, proteins were classified according to “Gene Ontology (GO) Biological Process” distribution option of String v11. The analysis was performed focused on mainly epigenetic modifiers.

## Results

### miRNAs identified to be overexpressed in Crohn’s diseases interact with mRNAs for enzymes associated with epigenetic and post-translational modifications: integrative analysis of miRNAs associated with CD

A total of 190 miRNAs were identified to be associated with CD (Additional file 1: Table S1), which were cross-indexed with 1,544 miRNAs that interact with mRNAs for enzymes related to epigenetic and post-translational modifications. This yielded 34 miRNAs that were present on both lists (Additional file 2: Table S2). From the 34 selected miRNAs, 26 were identified to interact with mRNAs for epigenetic modifiers (Additional file 2: Table S2).

**Fig. 1:**
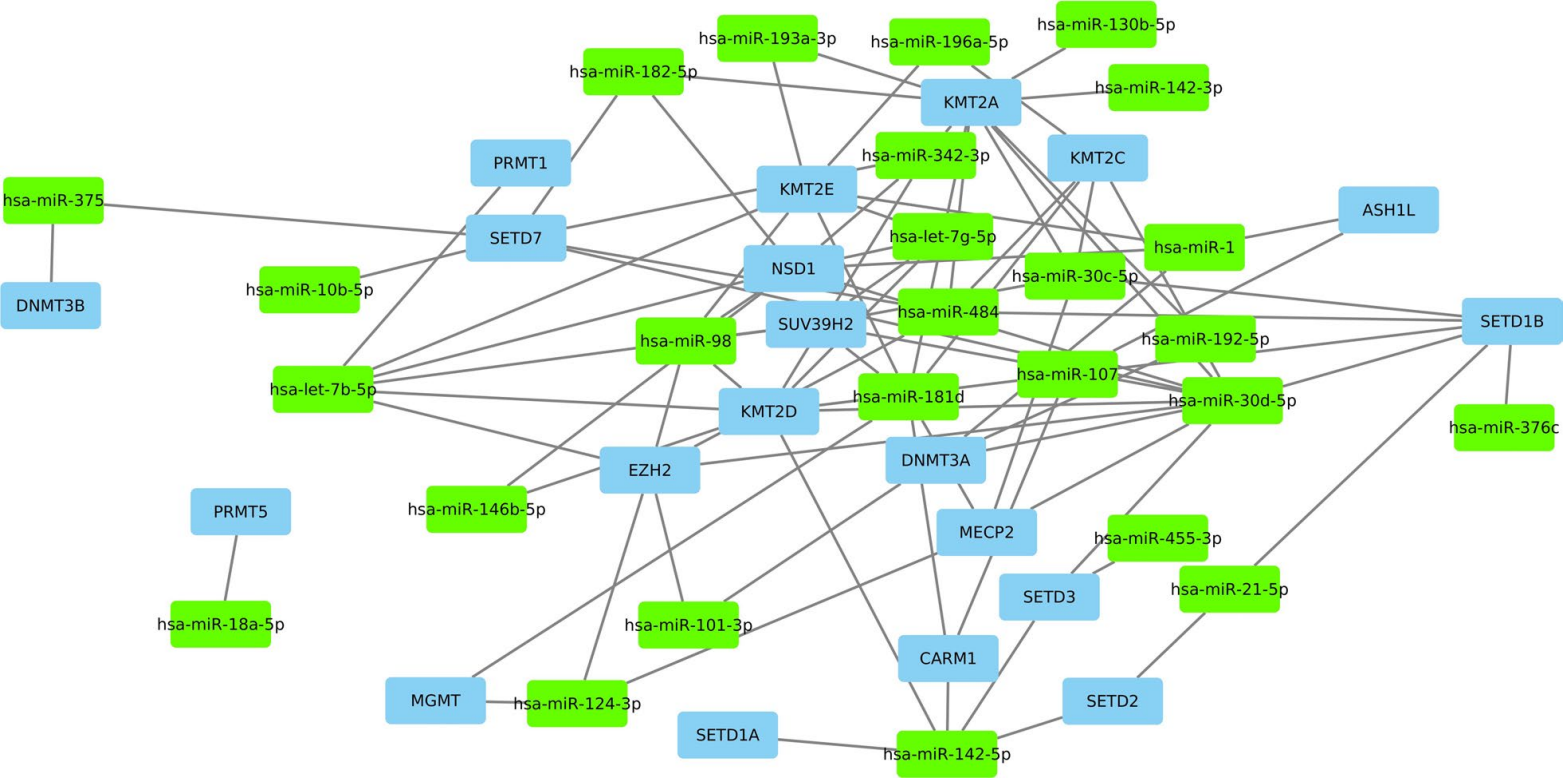
miRNA-Target mRNA network showing the interaction of miRNAs involved in Crohn's disease and their Target mRNA of proteins associated with epigenetic mechanisms. Green box nodes represent miRNAs and blue box nodes represent mRNAs

From the 190 miRNAs identified to be associated with CD, hsa-miR-30d-5p [3] regulates the largest number of mRNAs for enzymes associated with epigenetic and post-translational modifications, a total of 20 mRNAs: Ubiquitin-Conjugating Enzyme E2J 1 (UBE2J1) [62], Catechol-O-methyltransferase (COMT) [63], Ubiquitin-Conjugating Enzyme E2R 2 (UBE2R2) [64], Methil-CpG-Binding Protein 2 (MECP2) [65], DNA Methyltransferase 3A (DNMT3A) [66], Nuclear Receptor Binding SET Domain Protein 1 (NSD1) [67], UBE2D2, KMT2D, UBE2K, RNMT, MTR, KMT2C, SETD7, SETD3, UBE2D3, SETD1B, SUV39H2, KMT2A, CTLA4 and EZH2. In addition, hsa-let-7b-5p [68] regulates a total of 17 mRNAs: NSD1, CA12, UBE2D2, KMT2D, TRMT1, RNMT, KMT2E, NSUN2, SP1, UBE2A, UBE2Q1, UBE2D3, SUV39H2, TYMS, TRMT13, PRMT1 and EZH2, which are associated with epigenetic and post-translational modifications. Furthermore, hsa-miR-181d [69] downregulates 12 mRNAs associated with epigenetic and post-translational modifications: UBE2J1, UBE2W, MECP2, RNMT, KMT2C, KMT2E, SP1, UBE2D3, CARM1, SUV39H2, KMT2A, MGMT. Network shows the 26 miRNAs interacting with their target mRNAs associated with epigenetic modifiers (Fig. 1).

### Protein–protein interaction network of enzymes associated with epigenetic and post-translational modifications, whose expression is regulated by miRNAs involved in Crohn's disease

Biological interactions of identified enzymes associated with epigenetic and post-translational modifications, regulated by miRNAs involved in CD, were analyzed using String v11 database. From the 70 identified enzymes associated with PTMs, 63 show a protein–protein interaction network with 297 edges, and average node degree of 8.49, average local clustering coefficient of 0.654, and PPI enrichment *p* value < 1.0e−16.

**Fig. 2:**
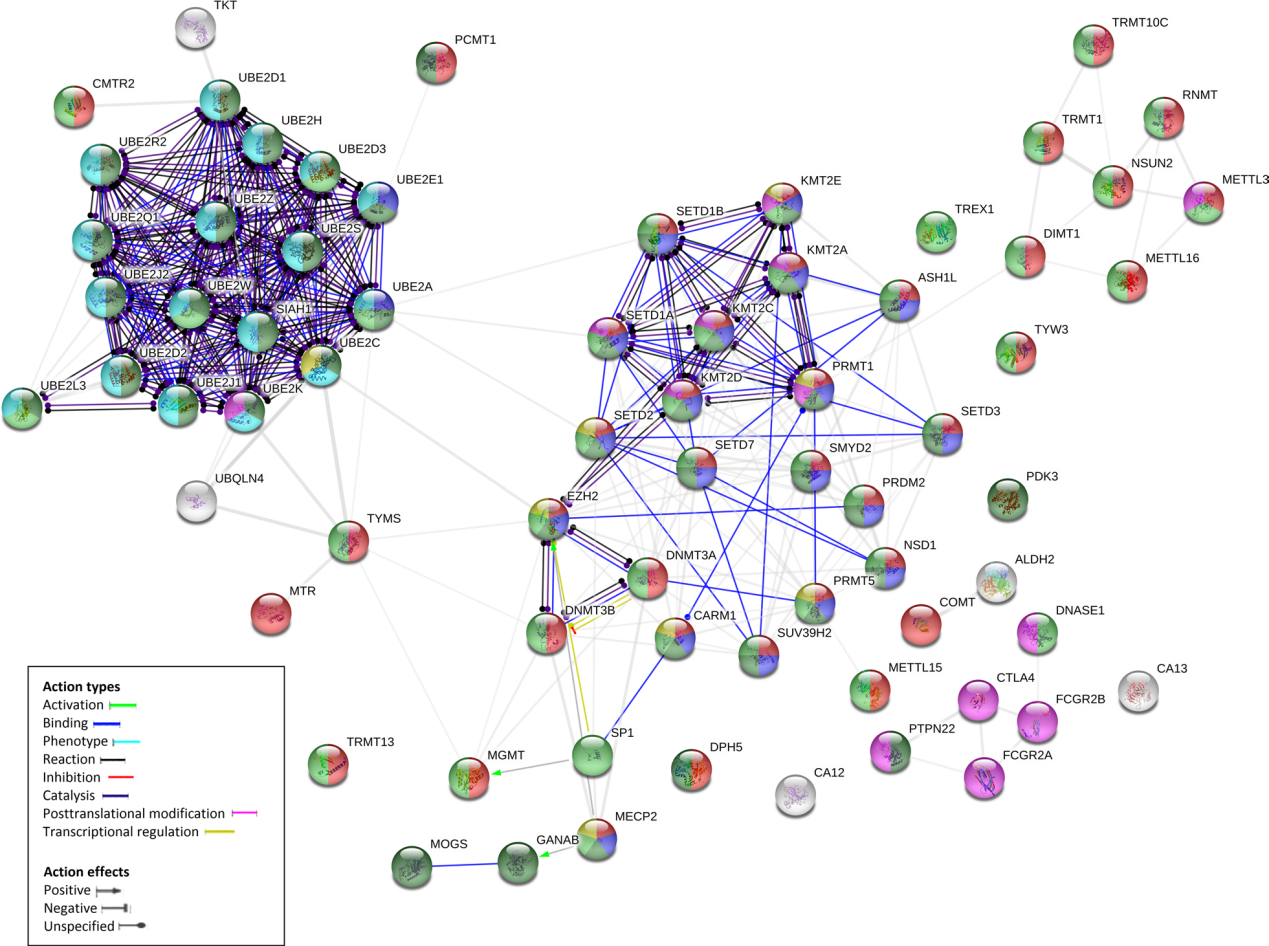
Functional interaction network of enzymes associated with epigenetic and post-translational modifications, whose expression is regulated by miRNAs involved in Crohn's disease. Proteins were classified by String v11 software according to “(GO) Biological Process” distribution option. Each node represents all the proteins produced by a single protein-coding gene locus. Red nodes correspond to proteins involved in methylation (GO:0032259), blue nodes to histone modification (GO:0016570), dark green nodes to protein metabolic process (GO:0019538), light green nodes to nucleic acid metabolic process (GO:0090304), aquamarine nodes to protein ubiquitination (GO:0016567), yellow nodes to regulation of cell cycle process (GO:0010564) and pink nodes to regulation of immune system process (GO:0002682), respectively. Edges represent protein–protein action type associations

According to biological processes, the proteins were classified as follows (Additional file 3: Table S3): 54,3 percent of proteins were related to methylation (GO:0032259) (TRMT10C, NSUN2, RNMT, METTL3, METTL16, TRMT1, DIMT1, TRMT13, DPH5, SETD3, ASH1L, NSD1, KMT2A, PRMT1, SMYD2, PRDM2, PRMT5, SETD7, KMT2C, KMT2D, KMT2E, SETD1B, SETD1A, SETD2, CARM1, SUV39H2, DNMT3A, EZH2, DNMT3B, MECP2, METTL15, MGMT, TYMS, MTR, COMT, TYW3, CMTR2, and PCMT1); 30% were related to histone modification (GO:0016570) (MECP2, SUV39H2, CARM1, EZH2, PRMT5, SETD7, SETD2, KMT2D, PRDM2, SMYD2, PRMT1, NSD1, SETD3, ASH1L, KMT2A, KMT2E, KMT2C, SETD1B, SETD1A, UBE2A and UBE2E1); 24,3% were related to protein ubiquitination (GO:0016567) (UBE2A, UBE2C, UBE2K, UBE2H, UBE2E1, UBE2S, UBE2D3, UBE2W, UBE2R2, UBE2D1, UBE2J2, UBE2D2, UBE2Z, UBE2J1, UBE2Q1, SIAH1 and UBE2L3); 11,4% were related to regulation of cell cycle process (GO:0010564) (KMT2E, PRMT1, SETD2, CARM1, EZH2, MECP2, UBE2C, PRMT5); 18,6% were related to regulation of immune system process (GO:0002682) (METTL3, DNASE1, FCGR2B, CTLA4, FCGR2A, PTPN22, PRMT1, KMT2A, KMT2E, KMT2D, SETD1A, KMT2C, UBE2K); 60% were associated with protein metabolic process (GO:0019538) (PCMT1, UBE2A, UBE2C, UBE2K, UBE2H, UBE2E1, UBE2S, UBE2D3, UBE2W, UBE2R2, UBE2D1, UBE2J2, UBE2D2, UBE2Z, UBE2J1, UBE2Q1, SIAH1, UBE2L3, MOGS, GANAB, MECP2, DPH5, SUV39H2, CARM1, PRMT5, EZH2, SETD7, PRDM2, NSD1, SMYD2, SETD2, KMT2D, PRMT1, SETD3, KMT2C, SETD1A, KMT2A, SETD1B, KMT2E, ASH1L, PDK3 and PTPN22) and 57,1% were related to nucleic acid metabolic process (GO:0090304) (CMTR2, UBE2L3, UBE2W, UBE2D3, UBE2A, TYMS, TRMT13, MGMT, DNMT3B, MECP2, SP1, DNMT3A, SUV39H2, CARM1, PRMT5, EZH2, SETD7, PRDM2, NSD1, SMYD2, SETD2, KMT2D, SETD3, KMT2C, SETD1A, KMT2A, SETD1B, KMT2E, ASH1L, TYW3, METTL15, TREX1, DNASE1, TRMT10C, TRMT1, NSUN2, RNMT, DIMT1, METTL3 and METTL16) (Fig. 2). Analysis allowed the identification of epigenetic modifiers (Additional file 3: Table S3).

## Discussion

The study of the interaction of miRNAs regulation pathways, mRNA targets and epigenetic mechanisms associated with diseases, can generate valuable knowledge about the subjacent molecular mechanisms of the pathologies and its chronicity, as well as the implication of genes variants in diseases. Furthermore, the identification of miRNAs profiles, strongly associated with diseases susceptibility and pathogenesis, could serve as a platform to the development of novel therapies with specific targets, and diagnostic markers to identify not only those affected, but those at-risk individuals prior to clinical onset of the disease [3, 8, 11, 68].

In the present work, we analyzed the gene expression profile regulated by identified miRNAs associated with CD. The analysis showed 190 miRNAs overexpressed in Crohn’s disease and their interactions with enzymes associated with epigenetic and post-translational modifications. From the identified 190 miRNAs associated with CD (Additional file 1: Table S1), 34 miRNAS were found to interact with mRNAS encoding enzymes that catalyze epigenetic and post-translational modifications (Additional file 2: Table S2), of which 26 interact with epigenetic modifiers (Fig. 1).

In addition, to explore the biological relevance of miRNAs in epigenetic mechanisms related to Crohn's disease, we analyze the landscape of miRNAs that have been shown experimentally greater relative abundance in patients with Crohn's disease and that interact with genes encoding enzymes that perform epigenetic modifications, since these mechanisms constitute ways of immunological, metabolic and cell cycle processes regulation (Fig. 2). Although the universe of mechanisms of action mediated by miRNAs is quite wide, here we associate a high expression of miRNAs with a decrease in the efficiency of the translation of miRNAs’ target mRNAs.

According to the data obtained, miRNAs identified overexpressed in CD associated with epigenetic mechanisms are involved in the regulation of several biological processes closely related to immunological and tumorigenic processes (Fig. 2). In this regard, dysregulation of Histone-lysine N-methyltransferase SUV39H2 (Suppressor of variegation 3–9 homolog 2) expression, whose encoding gene is a target of miRNAS upregulated in CD, hsa-let-7b-5p, hsa-let-7g-5p, hsa-miR-30c-5p, hsa-miR-30d-5p, hsa-miR-98, hsa-miR-181d (Fig. 1), has been evidenced to be involved in TGF beta-induced epithelial to mesenchymal transition, migration of cancer cells, carcinogenesis and metastasis [70]. It is interesting to mention that the role of TGF-beta in CD has been previously evidenced [71, 72]. TGF-beta is a regulatory cytokine secreted by intestinal epithelial cells and mucosal T cells that shows anti-inflammatory and protecting functions, decreasing local immune responses to luminal antigens, enhancing barrier function and promoting the production of secretory immunoglobulin A (IgA) [71, 72]. Thus, miRNAs dysregulation in CD could mediate epigenetic mechanisms implicated in TGF-beta signaling pathways thereby generating inflammation and carcinogenesis.

SUV39H2 downregulation has been also associated with autoimmune diseases. Decreased expression levels of SUV39H2 and reduction of global H3 lysine 9 methylation has been evidenced in CD4+ T cells from Latent autoimmune diabetes patients [73]. These data correlate with the miRNAs pattern of CD patients found in this study (Fig. 1). The upregulation of miRNAs whose target gene is SUV39H2 suggests a repression of SUV39H2 expression that can be involved in CD autoimmune pathogenesis.

Regarding T lymphocytes differentiation, Methyl-CpG binding protein 2 (MECP2), whose encoding gene expression is regulated by miRNAs found overexpressed in CD patients, such as hsa-miR-30c-5p, hsa-miR-124-3p, hsa-miR-30d-5p and hsa-miR-181d (Fig. 1), has been evidenced to bind methylated DNA, inducing transcriptional repression of target genes. Interestingly, MECP2 is essential for sustaining Foxp3 stable expression in Regulatory T cells (Tregs), as well as, Tregs lineage function during inflammation. Li et al. have described that in response to TCR activation and inflammatory cytokine signaling, conserved non-coding sequence 2 region (CNS2) of foxp3 locus recruits MECP2. MECP2 interacts with cAMP responsive element binding protein 1 (CREB1) and as a consequence rescues foxp3 transcription through local histone H3 acetylation. Thus, MECP2 induces in Tregs, resistance to inflammation-induced silencing of foxp3 via an epigenetic mechanisms [74]. In this regard, the high levels of miRNAs that regulate MECP2 encoding gene expression in CD could explain in part the phenotype of these patients and might be interesting targets in the generation of novel treatments for autoimmune and inflammatory diseases, including CD.

DNA methyltransferases (DNMTs) association with autoimmune diseases has been evidenced in patients affected by multiple sclerosis (MS). The DNA methylation status in immune cells and brain tissues from MS samples have shown alterations [75]. In the present study, miRNAs found upregulated in CD affected tissues, such as hsa-miR-192-5p, hsa-miR-30d-5p, hsa-miR-1, hsa-miR-101-3p and hsa-miR-375, are involved in gene expression regulation of DNMT3A, DNMT3B (Fig. 1). Increased levels of these enzymes have been described during CD4+ T cells polarization toward T helper 2 (Th2) phenotype compared with their expression in naïve or Th1 cells. DNMT3A has been also associated with DNA methylation at the Il13 locus, repressing Il13 expression in Th2 cells and thereby avoiding strong inflammatory allergic reactions [76]. Therefore, the regulation of DNMT3A expression by miRNAs in CD might be associated with CD4+ T cells polarization toward pro- inflammatory phenotypes, able to promote exacerbated inflammatory conditions.

Histone-Lysine *N*-Methyltransferases KMT2D, KMT2C, KMT2E, KMT2A are regulated by several miRNAs overexpressed in CD-affected tissues such as hsa-let-7b-5p, hsa-let-7g-5p, hsa-miR-146b-5p, hsa-miR-142-5p, hsa-miR-107, hsa-miR-342-3p, hsa-miR-30d-5p, hsa-miR-1, hsa-miR-30c-5p, hsa-miR-98, hsa-miR-196a-5p, hsa-miR-484, hsa-miR-130b-5p, hsa-miR-182-5p, hsa-miR-192-5p, hsa-miR-181d and hsa-miR-193a-3p (Fig. 1). KMT2 enzymes methylate H3K4 and this modification has been evidenced to have a role in tumorigenesis [77]. Low levels of KMT2D induce carcinogenesis via epigenetic modifications, repressing the expression of Period Circadian Regulator 2 (PER2) gene, which regulates tumor-promoting glycolytic genes [78]. According to these data, mutations in KMT2D gene have been identified in ulcerative colitis-associated colorectal neoplasia [79]. On the other hand, the Histone-Lysine *N*-Methyltransferase EZH2, whose gene expression is regulated by various miRNAS upregulated in CD, such as hsa-miR-484, hsa-miR-124-3p, hsa-let-7b-5p, hsa-miR-30d-5p, hsa-miR-98 and hsa-miR-101-3p (Fig. 1), plays an important role in maintaining intestinal barrier integrity and immune homeostasis in IBD through the regulation of TNFα signaling pathways, among other inflammatory molecules [80, 81]. Consistent with the upregulated miRNAs showed in the present study, EZH2 expression in IBD colonic epithelial cells has been found downregulated. Thus, the study of EZH2 regulation by miRNAs could generate a new target treatments aim to ameliorate the EZH2 expression in CD patients in order to preserve the intestinal epithelial health.

Additionally, Histone-Lysine *N*-Methyltransferases SETD2, SETD3, SETD7, SETD1A, SETD1B gene expression is regulated by miRNAs overexpressed in CD, such as hsa-miR-21-5p, hsa-miR-142-5p, hsa-miR-10b-5p, hsa-miR-30c-5p, hsa-miR-484, hsa-miR-376c, hsa-miR-342-3p, hsa-miR-455-3p, hsa-miR-30d-5p, hsa-miR-107, hsa-miR-182-5p and hsa-miR-375. These data correlate with the reduced level of SETD2 observed in IBD patients. Lack of SETD2 expression in intestinal tissues is associated with decreased number of mucus-producing goblet cells, immune cells infiltrate, high levels of pro-inflammatory cytokines and chemokines, loss of the mucosal barrier integrity and epithelium inflammatory injury that can progress toward high-grade dysplasia. These alterations are explained in part, by the SETD2 induced regulation of gene expression related to apoptotic pathways, inflammatory response and mainly with oxidative stress [82]. Thus, these data suggest that normal expression levels of SETD2 could avoid IBD pathogenesis mediating oxidative stress regulation via epigenetic mechanisms. Moreover, SETD7 downregulation has been associated with tumorigenesis mediated by epigenetic modifications [83, 84]. These findings show the relevance of Histone-Lysine N-Methyltransferases regulation by miRNAs overexpressed in CD, in IBD carcinogenesis and inflammatory processes.

Regarding to miRNAs found in this research, overexpressed in CD-affected mucosa, and associated with bacterial invasion of epithelial cells, such as hsa-miR-140-5p, hsa-miR-124-3p, hsa-let-7g-5p, hsa-miR-142-3p, hsa-miR-21-5p, hsa-let-7b-5p, hsa-miR-196a-5p and hsa-miR-182-5p, studies have demonstrated that gut microbiota composition could alter local miRNAs profile and epigenetic mechanisms, providing a suitable setting for IBD [85–87]. In recent years, it has been also described that miRNAs can mediate host-microbiota interactions and can affect the composition of gut microbiota [88]. Likewise, microbiota composition affects the DNA methylation pattern in intestinal epithelial cells [89]. Additionally, it is known that in neonates, the diversified and balanced microbiota composition is essential for the development and maturation of the immune system. The reviewed data support the evidence that the interaction of genetic factors, miRNAs, epigenetic modifications, immunological pathways and microbiota composition regulates the intestinal health. Thus, the dysbiosis, characterized by a decreasing in the prevalence of resident obligate anaerobic bacteria, and an increasing in relative abundance of facultative anaerobes, such as *Enterobacteriaceae*, found in the intestinal mucosa of CD patients, favors the production of metabolites that modulate signaling pathways involved in IBD pathogenesis and Colorectal carcinogenesis [86, 87, 89].

## Conclusion

The identification and analysis of miRNAs found overexpressed in CD-affected tissues, and their association with epigenetic mechanisms, have shown the influence of the interaction of miRNAs regulation, enzymes associated with epigenetic modifications and gut commensal microbiota composition, in CD pathogenesis processes, such as alterations in T cells polarization, cell cycle, carcinogenesis, oxidative stress and intestinal barrier integrity maintenance. These data shed light to the study and design of therapeutic approaches in CD, based in the regulation of tissue-specific miRNAs and enzymes associated with epigenetic modifications.

## Supplementary Information

**Additional file 1.** Summary of miRNAs indentified to be overexpressed in CD-affected tissues.

**Additional file 2.** **Supplementary Table 2**. Summary of miRNAS overexpressed in CD, whose target genes encode and proteins associated with epigenetic and post-translational modifications.

**Additional file 3.** **Supplementary Table 3**. Classification according to “(GO) Biological Process” of proteins associated with PTMs and epigenetics, whose expression is regulated by miRNAs overexpressed in CD.

## Abbreviations

IBD: Inflammatory bowel disease
CD: Crohn's disease
UC: Ulcerative colitis
miRNAs: MicroRNAs
PTMs: Post-translational modifications
HDACs: Histone deacetylase
HATs: Histone acetyltransferases
DNMTs: DNA methyltransferases
HMT: Histone methyltransferases
Tregs: Regulatory T cells
Th2: T-helper 2 cells
Th1: T-helper 1 cells
SNPs: Single-nucleotide polymorphisms
Foxp3: Forkhead box P3
